# The Age of Man

**Published:** 1859-12

**Authors:** 


**The Age of Man.**

M. Flourens once endeavored to calculate how long a man ought to live. And he made his reckoning by determining the duration of life in some of the lower animals, and finding how long it took for their skeleton to arrive at its perfect developement. He thus satisfied himself that man was a centenarian. But we know, alas! that in all the civilized countries of Europe the mean period of life does not exceed 35 or 40 years; that among the lower classes it may be as low as 80, and that among the upper classes it rarely exceeds 60 years. M. Hœser, a German professor, has, however, discovered a litte Oasis of the Blest, where the population's mean age reaches very near to the figure of M. Flourens. He relates that on one of the little hills which surround the Gulf of Naples, there exists a convent, called of the Cameldules, which is celebrated through the world for its picturesque position. The business of the pious inhabitants of it, consists wholly in prayer and silence. Their food is of the simplest, a purely vegetable diet.

"Their food the fruits, their drink the crystal well."

But a diet sufficient, says the learned professor, to repair the losses occasioned by labors so little fatigueing. "My guide," he relates, who looked like one of 40, was 70 years old: and he the youngest of the community. He assured me that the death of a Cameldule before the age of 90, was an unheard of event, and that a considerable number of the "religious exceeded 100 years of age." M. Hœser also makes out that men of genius, at all events, lived longer in ancient than in modern days. The age of Pericles in this respect beats all others. At Athens, the majority of citizens of that day attained the age of 80. Hippocrates was a specimen of this class. Xenophon and Sophocles reached to 80. Epicharmus to 97. Thales and Solon to 100, and Gorgias and Leontium to 108.—*Clinique Europeene.*

